# Associations between Polish school principals’ health literacy and implementation of the Health Promoting School approach during the COVID-19 pandemic

**DOI:** 10.1371/journal.pone.0301055

**Published:** 2024-04-02

**Authors:** Karina Leksy, Grzegorz Gawron, Rafaela Rosário

**Affiliations:** 1 Institute of Pedagogy, Department of Social Science, University of Silesia, Katowice, Poland; 2 Institute of Sociology, Department of Social Science, University of Silesia, Katowice, Poland; 3 School of Nursing, University of Minho, Braga, Portugal; 4 Health Sciences Research Unit: Nursing (UICISA: E), Nursing School of Coimbra (ESEnfC), Coimbra, Portugal; Kalasalingam Academy of Research and Education, INDIA

## Abstract

The coronavirus pandemic has contributed to increasing the responsibility of school principals for the health of all school community members. Moreover, evidence confirms the significant role of school principals’ health literacy (HL) for health promotion in schools. Therefore, the presented study aims to evaluate the associations between Polish school principals HL and the implementation of the Health Promoting School (HPS) approach in Polish schools. The present study was conducted as part of an international survey on the global COVID-HL network (www.covid-hl.eu) between June 2021 and December 2021. Three subscales of the HPS were considered and an exploratory analysis were used in this study. Associations between the median split of each subscale of HPS (outcome) and health literacy (predictor) were performed using logistic regression. Research results showed that the highest level of HPS implementation was directed at pupils. Principals perceived themselves as having the highest HL on the ‘accessibility’ subscale and these respondents had significantly higher odds of implementing learning opportunities for students. The study suggests that principals with adequate HL may be more likely to effectively implement HPS strategies in schools. This research could provide insights into the complex interplay between HL and the HPS approach and inform the development of more effective strategies for promoting health and HL in schools.

## 1. Introduction

Accepted at the 9th Global Conference for Health Promotion, the Shanghai Declaration recognises health and well-being as crucial to UN Sustainable Development Goals. It also confirms that health is a universal right, an essential resource and goal for everyday living, and a political priority for all countries. Thus, the Shanghai Declaration calls for intervention in this area and indicates health literacy (HL) (next to good governance, healthy cities, and social mobilisation) as one of four pathways to accelerate countries’ actions for health improvement [1]. HL has been recognized for a long time as an important factor both for understanding health information and prediction of health status [2]. Simultaneously, the post-pandemic crisis revealed many gaps in people’s knowledge, awareness, and related behaviours in health protection and promotion on individual and social levels [3, 4]. In other words, the coronavirus pandemic has proved the importance of HL and eHealth literacy (eHL), which can be seen as a significant weapon against the coronavirus-related infodemic and many other problems within public health.

Sentell indicated that both community and individual HL is essential for health, which is considerable in the context of people’s management and care (e.g., managers in the workplace, school leaders and teachers, parents, caregivers). Research suggests that community HL is critical to specific communities (e.g., school communities) [5, 6]. In turn, individual HL is a well-established predictor of personal health outcomes [7] and can simultaneously influence communities’ health. According to the WHO definition, HL ‘represents the cognitive and social skills which determine the motivation and ability of individuals to gain access to, understand and use information in ways which promote and maintain good health’ [8]. It encompasses action to improve personal and community health by changing individual lifestyles and living conditions. By improving people’s access to health information and their capacity to use it effectively, HL is also critical to empowerment [8]. Moreover, world digitalisation and the easy and fast spread of health-related information make eHL urgently needed. eHL merges health and media literacy and refers to ‘an individual’s ability to seek, understand and appraise health information from electronic resources and make informed health decisions for addressing a health problem in everyday activities’ [9]. In line with these definitions, we perceive acquiring skills in HL and eHL as a necessity and a universal right of entire communities, regardless of gender, age, and other sociodemographic differences. However, reliable health knowledge and awareness are in particular demand among decision-makers, managers, and other influential stakeholders who can commit effectively to health actions and interventions on health and its determinants [1].

One of the HL classifications was presented by Nutbeam [10, 11], who distinguishes: *basic/functional literacy*, *communicative/interactive literacy*, and *critical literacy*. *Basic/functional literacy* means ‘sufficient primary skills in reading and writing that enable effective functioning in an everyday situation (…)’ [10]. Within this level, improvement may include increasing people’s knowledge of health risks, available health services and adhering to recommended actions. Simultaneously, as a feature of traditional health education outcomes, it does not encourage people to communicate interactively and thus does not develop their HL skills and autonomy. *Communicative/interactive literacy* refers to ‘more advanced cognitive and literacy skills which, together with social skills, can be used to actively participate in everyday activities, to extract information and derive meaning from different forms of communication, and to apply new information to changing circumstances [10]. The most advanced level is *critical literacy* which is used to critically analyse information and, by this, ‘exert greater control over life events and situations’ [10]. As pointed out by Nutbeam, the two last types of this classification facilitate greater independence and individual empowerment.

Some researchers integrate HL with the Health Belief Model (HBM) [12–14], perceiving HL as a moderator of HBM. It is assumed that HL affects components of HBM, such as the perceived severity, perceived susceptibility, perceived benefits, perceived barriers, cues to action, and self-efficacy, as a catalyst [12]. Moreover, HBM is frequently recognized as an effective framework for designing educational interventions and promoting healthy behaviours, and considers behaviour as a function of the individual’s knowledge and attitude [15–17]. According to Mackert & Guadagno (2020), HL plays a role among HBM constructs in the following scope:

- the relationship between education and perceived severity, perceived benefits, and perceived barriers;
- the relationship between self-efficacy and behavior change;
- the role of media as a cue to action;
- the role of the individual education in the development of HL.

Awareness of the role of HL in the HBM will contribute to greater use of the HBM model in designing education interventions for individuals of all levels of health literacy [13]. Considering the context presented in this manuscript, we believe that school principals’ HL potentially influences HBM, which can then affect the implementation of the Health Promoting School (HPS) approach in schools. Therefore, HBM model is useful for school principals in designing special aspects for health promotion and educational interventions [12, 18].

Nowadays, it is highly desirable to improve peoples’ HL, since it has turned out that more than a third of the worldwide population has difficulties finding, understanding, evaluating, and using information necessary to manage their health [19, 20]. However, reliable health information should be easily accessible and understandable for all. Therefore, building a health-promoting education system that consists of intentional, planned actions institutionalising health promotion in all its functions is crucial [21].

Despite the wide range of people involved in school health promotion (pupils, teacher, non-teaching staff, parents), school principals are increasingly identified as the main actors for initiating and sustaining standardised and complex interventions in school health promotion [22–26]. Scientific evidence confirms the significant role of school principals’ knowledge, understanding, competencies, and motivation for health promotion. Their engagement and positive attitude to health matters facilitate and support the realisation of the whole school approach to health promotion. For example, according to the research results from the Norwegian Network on Health Promoting Schools, the positive attitude of school principals towards school health promotion was vital for running and maintaining the programme in schools [27]. Similarly, research evidence from an Austrian case study suggests school principals were mostly initiators of school health promotion and in deciding concrete health-promoting activities [25]. Moreover, according to Kam et al. [23], high school principal support was associated with improving pupils’ health-related behaviours.

The coronavirus pandemic has ‘imposed’ on school leaders an even more significant commitment to health promotion in school [28]. We assume that principals with a higher level of HL, genuinely involved in health promotion at school before the pandemic, coped better during the pandemic. This assumption aligns with the German results, which showed that limited HL among male school leaders was associated with low levels of health-promoting school activities [22]. During the pandemic, school principals’ HL especially played a crucial role due to the amount of health-related information that school leaders had to face [29].

The previous research was not conducted under the conditions of a pandemic on the scale of Covid-19. Therefore, there has been scant research about the role of school principals’ HL and its association with implementing the Health Promoting School (HPS) approach. For example, Dadaczynski et al. [22] conducted a study investigating the level of health literacy among school leaders and its association with the extent of HPS implementation. Simultaneously, it must be emphasized that during the pandemic, school principals’ influence on students’ health and healthy behaviours was significantly limited due to isolation and limited physical access to pupils. Although the Polish survey confirmed that schools belonging to the HPS network implemented more effective health promotion principles in schools during COVID-19 [30]. Still, the possibility of realizing school health promotion politics in extended school closure was difficult. Moreover, in Poland, school principals were highly focused on ensuring continuity of education in conditions of social isolation [31, 32] and the problem of ’disappearing students’ [33, 34]. At that time, it was a real threat that ’the whole-school approach to health might be reduced to behavioural (e.g., hygiene-related) approaches’ [28]. Moreover, schools seem to have put more effort into core subjects, while health promotion matters received less attention [28].

Despite this growing body of research, there remains a gap in evidence pertaining to the health literacy of Polish school principals as a potential determinant in facilitating the adoption of the HPS approach, particularly in the context of the COVID-19 pandemic. We intend to overcome this issue, aiming to evaluate the associations between Polish school principals’ HL in relation to the implementation of the HPS approach in Poland in the middle phase of the pandemic. Thus, in the presented study, two main research questions were established:

1. Is it the association between Polish school principals’ HL level and HPS implementation?
2. Which of the Polish school principals’ HL dimensions was most associated with HPS implementation during the pandemic?

We hypothesise that school principals’ high level of HL is positively associated with implementing the HPS approach during the coronavirus pandemic. We also predict that school leaders with higher HL for ‘accessibility’ and ‘understading’ have higher odds of implementing the HPS approach in their schools.

## 2. Research methodology

### 2.1. Study design and data collection

The present study was conducted as part of an international study on the global COVID-Health Literacy Research Network (www.covid-hl.eu). In the initial phase of the research, we randomly selected eight out of sixteen voivodeships (Śląskie, Podkarpackie, Podlaskie, Kujawsko-Pomorskie, Łódzkie, Warmińsko-Mazurskie, Wielkopolskie, Lubuskie). After that, we sent the information about the planning survey to the Regional Education Boards with a request to disseminate the questionnaire to the primary and secondary schools in their regions. One of six Regional Education Boards (representng Silesian region) agreed to distribute the survey link in the region’s schools. At the same time, five Regional Education Boards only approved the research, advising researchers to use the ’List of schools and educational institutions’ (Wykaz szkół i placówek oświatowych—Otwarte Dane). In consequence, researchers sent the invitation to the school principals’ survey via email. The link with the survey was sent to primary and secondary schools, excluding special schools. In the case of special schools, we assumed that the work-related demands of principals working in these schools differed significantly during the pandemic, and burdens for school leaders were probably much higher. Consequently, over 10 thousand invitations were sent to primary and secondary school principals. The survey was conducted online between June 2021 and December 2021. The research tool used in this survey (and all other country surveys within the COVID-HL Research Network) was developed by Dadaczynski et al. [35] and translated into Polish. In the survey instruction, participants were informed about the study’s purpose, voluntary nature, and anonymity. The instructions also state that joining the survey means the participant’s conscious consent to participate in the research. Simultaneously, respondents were informed that they might stop completing the survey at any time or resign from participating without any consequences. A total of 1899 Polish school principals took the survey (18% of questionnaire returns), within which 832 completed the whole questionnaire. Simultaneously, the research sample for individual questions differs because the respondents answered only some of the survey’s questions. Concerning questions presented in this manuscript, the sample size for HPS implementation (Q25) questions was 852 for HPS-students, 850 for HPS-teaching and 843 for HPS-principles. At the same time, the sample size for the HLS-Covid-Q22 subscales was respectively: access– 858; understand– 860; appraise– 850; apply– 862.

### 2.2. Description of the structure of the research sample

**Fig 1:**
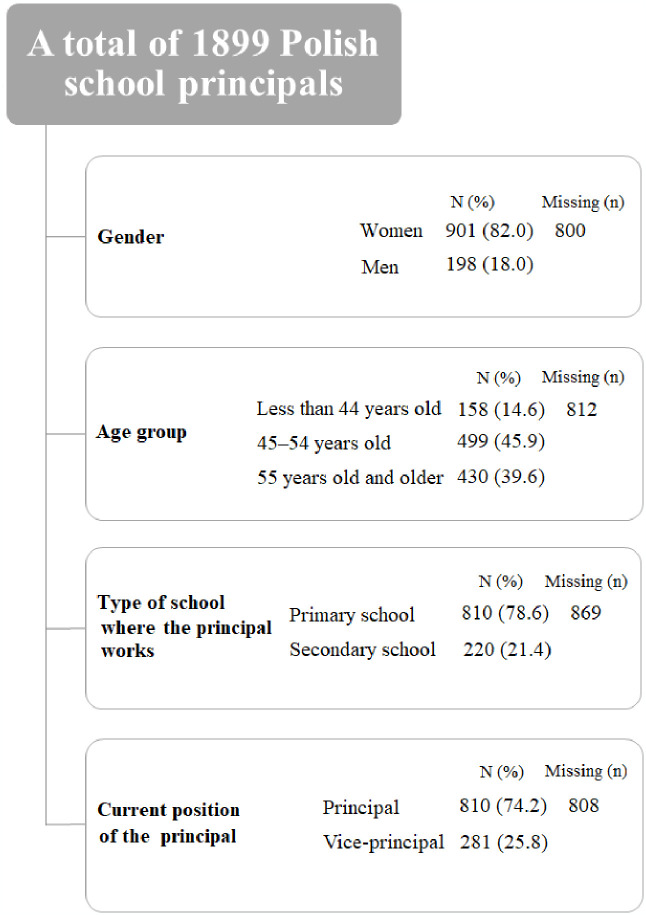
The structure of the research sample.

In the presented study, most survey participants were women (82.0%), which probably comes from the fact that in the Polish educational system, the teaching profession is more common among women than men [36, 37]. Most respondents were 45–54 years old (45.9%) and representing primary schools (78.6%). Almost half (49%) were principals of HPSs (please see Fig 1).

### 2.3. Measurements

The tool used in the presented study (COVID-19 Health Literacy School Principals Survey) consists of five parts: 1- Sociodemographic information; 2- Current work situation; 3- Health information in the context of the COVID-19 pandemic; 4- Health Promoting Schools (HPS) during the COVID-19 pandemic in Poland; and 5- Health situation. Each part consists of several specific aspects. In the manuscript, we presented research results from health literacy questionnaire and HPS.

Regarding the operationalization of **HPS**, a self-developed scale based on previous work developed by Dadaczynski et al. [38], was adjusted to the coronavirus pandemic [35]. Originally the HPS scale consisted of 15 items, but in the Polish adaptation, we merged two items (‘At our school, health-promoting aspects play an important role in the design of teaching and learning conditions [including homeschooling]’, and ‘At our school, health-promoting aspects play an important role in the design of working conditions [including home office]’) assuming that during the coronavirus pandemic, these aspects of daily life, such as working and learning, were happening at home simultaneously. As a result, we analysed 14 items on this scale. Respondents assessed each item on a 4-point scale concerning their agreement with each statement (1 –not true at all; 2 –mostly not true; 3 –likely to be true; 4 –totally true). This scale has three subscales: covid-19 related support for students (HPS-students, e.g., item 3 "At our school students learn how to get enough exercise despite the restrictions due to coronavirus"); teaching, learning and working conditions (HPS-teaching, e.g., item 6 "At our school, school staff are supported in dealing with stressful situations caused by coronavirus (e.g., stress”); and principles of HPS (HPS-principles, e.g., item 11 „at our school we work closely with community stakeholders from the health and social sectors when it comes to promoting and protecting the health of our students"). Exploratory factor analysis showed satisfactory indices of chi-square (chi-square (52) = 98.0, p < .001), the comparative fit index (CFI) = 0.996, the Tucker-Lewis index (TLI) = 0.994, and the root mean square error of approximation (RMSEA) = 0.067.

In this sample, the overall Cronbach’s alpha was 0.954, and McDonald’s omega was 0.954. The Cronbach’s alpha for HPS-students was 0.933, with McDonald’s omega at 0.932. For HPS-teaching, the Cronbach’s alpha was 0.895, and McDonald’s omega was 0.893. In the case of HPS-principles, the Cronbach’s alpha was 0.894, and McDonald’s omega was 0.894. A mean value was calculated for each subscale. For further analysis, two subgroups were created using the median split (0 = low level of HPS and 1 = high level of HPS).

The **coronavirus-related HL questionnaire (HLS-COVID-Q22)**, based on the European Health Literacy Survey Questionnaire (HLS-EU-Q) [9] and developed by Okan et al. [39], was used to assess Polish school principals’ HL during the pandemic. The questionnaire consisted of 22 items grouped into four subscales: accessing (six items), understanding (six items), appraising (five items), and applying (five items) health-related information in relation to the coronavirus pandemic. Each of the 22 items started with: ‘On a scale from very easy to very difficult, how easy would you say it is to …’. Respondents answered on a 4-point scale ranging from 1 –very difficult to 4 –very easy. Sample questions for each subscale within the HLS-COVID-Q22 are (respectively): ‘… find information about the coronavirus on the internet?’; ‘… understand the risks of coronavirus that I find on the internet?’; ‘… judge how much I am at risk of coronavirus infection?’; ‘… behave in a way to avoid infecting others?’. The survey participants answered on a 4-point scale (1 = very easy, 4 = very difficult). The internal consistency for each subscale was 0.89 (accessing), 0.91 (understanding), 0.88 (appraising), and 0.92 (applying).

Additionally, sociodemographic characteristics, including gender, age (less than 44, 45–54, 55 and older), and the type of school where school principals worked (primary or secondary), were gathered.

### 2.4. Statistical analysis

We developed descriptive statistics as means and standard deviations (SD) and percentages according to the type of variables. The internal consistency reliability study was done through analysis of the Cronbach’s alpha and McDonald omega [40–43].

Associations between the median split of each subscale of HPS (outcome) and health literacy (predictor) were performed using logistic regression. An adjusted odds ratio (OR) with a 95% confidence interval (CI) was considered. This form of regression analysis was chosen because there are no predefined cut-off values for the outcome variable resulting in an empirical division of sufficient versus limited health promotion practices. As potential confounders, we included the variables of gender, age, type of school (primary/secondary), and belonging to the HPS network. The data analysis was performed using SPSS, version 29.0 (SPSS Inc., Chicago, IL), with a 0.05 level of significance.

## 3. Results

Table 1 provides the descriptives of the participants. The majority of the participants were principals (74.2%), belonging to primary schools (78.6%). Across the four domains of HL ‘appraising’ was perceived as the most difficult (mean of 3.0, SD of 0.6), while ‘accessibility’ was perceived as the easiest (mean of 3.4 and SD of 0.5).

**Table 1:** The descriptive results of health literacy and health promotion practices of principals.

	Mean (SD)	
**Health literacy (HL)**		
HL–accessibility	3.4 (0.5)	1041
HL–understanding	3.2 (0.6)	1039
HL–appraising	3.0 (0.6)	1049
HL–applying	3.2 (0.6)	1037
**Health promotion practices (HPP)HPS**		
HPP HPS—students–students	3.6 (0.6)	1047
HPS–stakeholders teaching	3.3 (0.6)	1049
HPS—principles	3.3 (0.6)	1056

Compared with the dimension of ‘HPS-students’ (M = 3.6, SD = 0.6), ’HPS-teaching’ and ’HPS-principles’ were perceived as having lower implementation (M = 3.3, SD = 0.6) (please see Table 1).

Each dimension of HL was significantly associated with HPS. In the adjusted model, principals with adequate HL had significantly higher odds of implementing HPS-students ranging from HL-appraising (aOR = 2.0, 95% CI = 1.5; 2.7) to HL-accessibility and HL-applying (aOR = 2.2, 95% CI = 1.6; 2.9 and aOR = 2.2, 95% CI 1.6; 2.9). HPS-teaching was 2.4 times more likely to be implemented by those principals with adequate HL understanding (95% CI 1.8; 3.2) and HL applying (95% CI 1.8; 3.3). Simultaneously, HPS-principles were 2.2 times more likely to be implemented by those principals with adequate HL understanding (95% CI 1.7; 3.0) and HL applying (95% CI 1.7; 3.0) (please see Table 2).

**Table 2:** Associations between HPS implementation and health literacy.

Health literacy	Health promoting schools (HPS) OR (95% CI)					
	HPS—students		HPS-teaching		HPS-principles	
	Crude	Adjusted	Crude	Adjusted	Crude	Adjusted
HL–accessibility	**1.9 (1.4; 2.5)**	**2.2 (1.6; 2.9)**	**2.1 (1.6; 2.7)**	**2.2 (1.6; 2.9)**	**1.9 (1.4; 2.5)**	**2.0 (1.5; 2.7)**
HL–understanding	**1.9 (1.4; 2.5)**	**2.1 (1.6; 2.9)**	**2.2 (1.7; 3.0)**	**2.4 (1.8; 3.2)**	**2.1 (1.6; 2.8)**	**2.2 (1.7; 3.0)**
HL–appraising	**1.7 (1.3; 2.3)**	**2.0 (1.5; 2.7)**	**2.1 (1.6; 2.8)**	**2.3 (1.7; 3.1)**	**1.5 (1.2; 2.0)**	**1.6 (1.2; 2.1)**
HL–applying	**1.7 (1.3; 2.3)**	**2.2 (1.6; 2.9)**	**2.2 (1.6; 2.9)**	**2.4 (1.8; 3.3)**	**2.0 (1.5; 2.6)**	**2.2 (1.7; 3.0)**

## 4. Discussion

The current study shows significant associations between Polish school principals’ HL related to COVID-19 and HPS in all dimensions. This issue is crucial as few scales are holistically adapted to school health promotion strategies [38, 44]. In addition, we are not aware of any study developed in Poland that addresses this relevant field. Simultaneously, according to existing evidence, leadership plays a vital role in developing and implementing HPS intervention strategies [22, 38]. This is especially important in those educational systems and school settings where health education and promotion are not systematically realised, and large latitude in this context is allowed. Research results from Taiwan with n = 1,140 school principals and n = 1,110 HPS coordinators indicate that school leaders’ understanding of the HPS approach and their willingness to sustain HPSs in their schools were significantly associated with higher levels of HPS implementation [26].

Our research results showed that principals perceived that the highest level of HPS implementation was directed at students. This result seems to confirm that students are the first and most important ‘beneficiaries’ of the school, and their safety and health protection is the priority.

According to the presented research results, principals perceived themselves as having the highest HL on the ‘accessibility’ subscale. This result may have stemmed from the wide and easy access to health-related information in the context of the pandemic. However, we can question how credible this information was in the face of the ubiquitous infodemic [3, 45]. In the face of lots of unchecked published information [39, 45, 46], especially in digital media, critical assessment and competencies to understand it correctly were needed, particularly for those who were responsible for others’ health and life [47]. Thus, we assume that many school principals had to ‘navigate the complex information environments marked by high levels of uncertainty in order to remain healthy and take relevant precautions using the information available’ [45]. For them, adequate HL was probably one of the primary conditions for compliance with government regulations and recommendations [4].

Moreover, those principals with higher HL for ‘accessibility’ had significantly higher odds of implementing HPS-students (aOR = 2.2, 95% CI 1.6; 2.9) and HPS-teachers (aOR = 2.2, 95% CI 1.6; 2.9). We can assume that it was connected with the willingness of school principals to search for and use coronavirus-related information from different sources. It is also possible that participants’ attitudes to health and health promotion in schools is relevant. The HL for “understanding” (aOR = 2.2, 95% CI 1.7; 3.0) and “applying” (aOR = 2.2, 95% CI 1.7; 3.0) was significantly associated with HPS-principles. As Betschart et al. [48] indicated, attitudes toward health promotion amplified the positive relationship between HL and HPS approach. Specifically, when positive attitudes and understanding toward health promotion occurs, school principals’ HL is significantly associated with HPS. Also, it is suggested that better knowledge of the holistic school approach concept and awareness of the importance of the active engagement of the whole school community in health promotion and protection. Forging stronger ties with parent/community groups to support families, teenage and children’s health and well-being is now a must [30, 49, 50]. Concerning the research results presented in this paper, it seems that the HL of Polish school principals is significantly associated with a higher odd of implementing the HPS approach during the COVID-19 pandemic.

Interestingly, the school principals’ lowest HL was on the ‘appraising’ subscale, not only as coefficients in association with HPS, but also as mean values. It seems that, overall, judging health information is very challenging. Also, studies from Germany and Portugal confirmed that even people who had a satisfactory level of HL/eHL demonstrated difficulties in assessing whether they could believe in health information found online and on social media [39, 51]. Referring to the result obtained for the Nutbeam’s HL classification, this suggests that *critical health literacy* is the most difficult to achieve. This complexity arises from the need to acquire skills for critically evaluating health information from diverse sources, including the comprehension of various health determinants such as social, economic, and environmental factors. Moreover, critical health literacy is intricately associated with broader population welfare, extending beyond individual-level advantages [52]. Therefore gaining of critical HL by school principals can be crucial to both population (the members of the whole school community) and individual (pupil, teacher, non-teaching staff member) and may result in improving peoples’ capacity to act for health and its determinants [10].

Developing critical health literacy among school principals is vital as it is connected to contemporary health promotion models. A high level of health literacy can provide greater autonomy and control over health decision-making [53] and improve individual and community empowerment [40]. Moreover, health-related information needs to be adjusted to better understand the broad range of determinants of health, both personal and societal [52], especially in the school context. Given the data obtained, we believe improving school principals’ HL is crucial for effectively implementing HPS supporting students, teaching, working conditions and principles. Simultaneously, the HL of school principals can be increased, among others, by including content related to school health promotion during school principals’ initial and in-service training [54, 55]. It is also essential to develop a health-promoting leadership model of school managing, understood as ’[…] leadership that is concerned with creating a culture for health-promoting workplaces and values to inspire and motivate the employees to participate in such a development’ [56]. This leadership model requires genuine interest and engagement of school leaders in individual and school community health. In this context, it is essential to develop not only personal but also organizational HL. Organisational HL provides a framework on the systems level for implementing a whole-school approach to promoting health literacy, including all school community members [57]. ‘Schools as educational institutions have the potential to address individual differences in learning and to narrow the disparities in learning caused by disadvantaged backgrounds’ [58].

## 5. Summary

The presented research results exhibit several significant limitations. Firstly, it was a cross-sectional study conducted in the midst of the pandemic (June–December 2021). Secondly, the sample size is not representative for the whole country since school principals from eight voivodeships took part in the study. Thirdly, the study results rely on the self-assessment of the respondents regarding Health Literacy (HL) and declarations about Health-Promoting Schools (HPS) implementation during COVID-19. Consequently, there may be other dimensions of HL beyond those included in this study that could impact the implementation of HPSs. Nonetheless, the presented research contributes to drawing attention to the significance of school principals’ HL, particularly for the critical literacy type, which appears to be most effective in the context of health protection and promotion. However, this study has also strengths. First, the study included almost 1,900 principals, which is a sizeable sample of the population being studied. Second, the majority of participants were women and aged 45–54 years, which is a demographic often underrepresented in research. Including this group can provide valuable insights into the experiences and perspectives of a diverse population. Third, the study included principals from primary schools and HPSs, which can provide a comprehensive understanding of the factors influencing health promotion in schools.

## 6. Conclusions

The current study yielded significant associations between principals’ HL and the implementation of the HPS approach, which adds to our understanding of the connection of these concepts in practice. The study suggests that principals with adequate HL may be more likely to effectively implement HPS strategies in their schools. Further research is needed to identify contextual issues in other dimensions of HL and implementation of the HPS approach. Such research could provide insights into the complex interplay between HL and HPSs and inform the development of more effective strategies for promoting health and HL in schools.

## Supporting information

S1 Appendix (PDF)

S2 Appendix (ZIP)
