# Supplementary material for: Associations between Polish school principals’ health literacy and implementation of the Health Promoting School approach during the COVID-19 pandemic
Source: PLoS One. 2024 Apr 2;19(4):e0301055. doi: 10.1371/journal.pone.0301055 (PMC10986982; doi:10.1371/journal.pone.0301055)
Supplement: S1 Appendix — (PDF) [file pone.0301055.s001.pdf]

Let`s start by asking you some information about yourself and the school you work at.

1. Please indicate your sex.

☐ Male ☐ Female ☐ Other

2. How old are you?

______ years of age

3. Please indicate the type of school in which you work. [please adapt to your country]

- ☐ Primary school
- ☐ Integrated secondary school
- ☐ Secondary middle school
- ☐ Comprehensive school
- ☐ Secondary school
- ☐ Grammar school
- ☐ Schools for children with special educational needs
- ☐ Vocational school
- ☐ Other

4. What is your current position at your school?

- ☐ School principal
- ☐ Member of the school management board (e.g., vice principal)

5. What is your weekly teaching load?

Please indicate the number of school hours per week.

_________________ hours/week

6. How many hours do you currently work on average per week?

Includes management & leadership asks, preparation and follow-up, class time, correction, extracurricular activities, etc.

_________________ hours per week

7. Please compare your current weekly work hours with those before the first measures were taken in schools due to the COVID-19 pandemic in March 2020. My current weekly work hours are ...

- ☐ Lower than before the COVID-19 pandemic
- ☐ About the same
- ☐ Higher than before the COVID-19 pandemic

8.	How many students attend your school?
	_______________ students

9.	What percentage of students in your school come from families from ... (max. 100 percent)	
	...rather low social classes	______ percent
	...more middle social classes	______ percent
	...rather higher social classes	______ percent

10. Does your school participate in the state programme Education and Health? [Education and Health is a state health programme, please adapt to your country]

- ☐ For less than < 1 year
- ☐ For 1 to < 2 years
- ☐ For 2 to < 3 years
- ☐ For 3 or more years
- ☐ No

11. Does your school use a specific media literacy curriculum and action plan that regulates the use of digital media (e.g. equipment, software) as well as the teaching of digital education and literacy?

- ☐ Yes, based on [please include national or regional media literacy curriculum from your country]
- ☐ Yes, based on [please include national/regional action plans on media literacy from your country]
- ☐ Yes, based on ____
- ☐ No, not in place

12. Does your school have ...

	Yes, a sufficient number	Yes, but not a sufficient number	No
... laptops as a permanent media pool in the classrooms?	☐	☐	☐
... laptops as a mobile media pool, e.g. on laptop trolleys?	☐	☐	☐
... tablets as a permanent media pool in the classrooms?	☐	☐	☐
... tablets as a mobile media pool, e.g. on tablet trolleys?	☐	☐	☐
... smartboards in classrooms?	☐	☐	☐
... projectors as a permanent feature of classrooms (ceiling projectors)?	☐	☐	☐
... mobile projectors?	☐	☐	☐

13.	**How would you rate the (digital) media equipment and infrastructure at your school overall regarding the following aspects?** Please rate from 1 (very good) to 6 (very bad).

The adequacy of the IT and media infrastructure to meet the requirements of COVID-19 related home-schooling	__________ (please enter your assessment here)
The competencies and skills of the school staff to handle the IT and media infrastructure	__________ (please enter your assessment here)
The quality and stability of the Internet connection at your school	__________ (please enter your assessment here)

The following is an assessment of your current work situation and how you deal with work-related demands.

14.	**How do you personally find your current job respectively work situation in general?** Put a cross in each row at the point which best corresponds with your feelings.

manageable	☐	☐	☐	☐	☐	☐	☐	unmanageable
meaningless	☐	☐	☐	☐	☐	☐	☐	meaningful
structured	☐	☐	☐	☐	☐	☐	☐	unstrctured
easy to influence	☐	☐	☐	☐	☐	☐	☐	impossible to influence
insignificant	☐	☐	☐	☐	☐	☐	☐	significant
clear	☐	☐	☐	☐	☐	☐	☐	unclear
controllable	☐	☐	☐	☐	☐	☐	☐	uncontrollable
unrewarding	☐	☐	☐	☐	☐	☐	☐	rewarding
predictable	☐	☐	☐	☐	☐	☐	☐	unpredicting

15.	**The questions in this scale are about your feelings and thoughts regarding your work during the COVID-19 pandemic in last month.** Please indicate how often you felt or thought a certain way **due to the COVID-19 pandemic**. **In the last month, …**

	Never	Almost never	Some-times	Fairly often	Very often
…how often have you been upset because of something that happened unexpectedly at your work at school?	☐	☐	☐	☐	☐
…how often have you felt that you were unable to control the important things at your work at school?	☐	☐	☐	☐	☐
…how often have you felt nervous and “stressed” at your work at school?	☐	☐	☐	☐	☐
…how often have you felt confident about your ability to handle your professional work-related problems caused by the COVID-19 pandemic?	☐	☐	☐	☐	☐
…how often have you felt that things at your work at school were ‘going your way’?	☐	☐	☐	☐	☐
…how often have you found that you could not cope with all your work tasks at school?	☐	☐	☐	☐	☐

…how often have you been able to control irritations at your work at school?	☐	☐	☐	☐	☐
…how often have you felt that you were on top of things at your work at school?	☐	☐	☐	☐	☐
…how often have you been angered because of things at your that were outside of your control at your work at school?	☐	☐	☐	☐	☐
…how often at your work at school have you felt difficulties were piling up so high that you could not overcome them?	☐	☐	☐	☐	☐

16. In the last three months, how often has it happened due to the COVID-19 pandemic that you had to …

	Never/ very rarely	Rarely	Some-times	Often	Very often
…be available for your colleagues as well as pupils and parents in your free time?	☐	☐	☐	☐	☐
…give up leisure activities in favour of work?	☐	☐	☐	☐	☐
…forgo getting sufficient sleep in favour of work?	☐	☐	☐	☐	☐
…work extra hours in your free time (after work, during holidays, at weekends, on public holidays)?	☐	☐	☐	☐	☐
…waived breaks (short breaks or lunch break) during your working hours?	☐	☐	☐	☐	☐
…worked longer than contractually agreed?	☐	☐	☐	☐	☐

17. In the past three months, how often has it happened that you have been working at a pace due to the COVID-19 pandemic that ...

	Never/ very rarely	Rarely	Some-times	Often	Very often
…you find burdensome?	☐	☐	☐	☐	☐
…you cannot sustain in the long term?	☐	☐	☐	☐	☐
…you know is not good for you?	☐	☐	☐	☐	☐

18. In the past 3 months, how often have you been required by the COVID-19 pandemic to …

	Never/ very rarely	Rarely	Some-times	Often	Very often
…be satisfied even with a less good work result than you would normally be?	☐	☐	☐	☐	☐
…lower your own work output standards somewhat	☐	☐	☐	☐	☐
…do some more superficial work	☐	☐	☐	☐	☐

19. If you now think about everything that plays a role in your work (e.g. the job, the working conditions, the students, the working hours, etc.). How satisfied are you with your work overall?

- ☐ Very dissatisfied
- ☐ Quite dissatisfied
- ☐ Neither satisfied nor dissatisfied
- ☐ Quite satisfied
- ☐ Very satisfied

In the following, it is all about how easy or difficult it is for you personally to deal with information around the topic of the coronavirus and COVID-19.

20. How well informed do you feel about the coronavirus or the corona pandemic?

- ☐ Very informed
- ☐ Good
- ☐ Satisfactory
- ☐ Poor
- ☐ Insufficiently informed

21. You still read and hear a lot about the coronavirus. How do you feel about it: Do you feel confused about COVID-19 information?

- ☐ Not at all confused
- ☐ A little confused
- ☐ Quite confused
- ☐ Very confused

22. Will you get vaccinated against coronavirus (COVID-19) if you are offered a vaccination?

- ☐ Yes, certainly
- ☐ Likely
- ☐ Maybe
- ☐ Unlikely
- ☐ Certainly not

23.	On a scale from 'strongly agree' to 'strongly disagree', how much do you agree with the following statements?			
	Totally agree	Agree	Not agree	Not agree at all
Vaccinations are important to protect myself and my family.	☐	☐	☐	☐
Overall, I believe that vaccinations are safe.	☐	☐	☐	☐
Overall, I believe that vaccinations are effective	☐	☐	☐	☐
Vaccination is compatible with my attitudes or religious beliefs.	☐	☐	☐	☐

24.	On a scale from very easy to very difficult, how easy would you say it is to…			
	Very easy	Easy	Difficult	Very difficult
… find information about the coronavirus on the internet?	☐	☐	☐	☐
… find information on the internet about protective behaviours that can help to prevent infection with the coronavirus?	☐	☐	☐	☐
… find information in newspapers, magazines and on tv about behaviours that can help to prevent infection with the coronavirus?	☐	☐	☐	☐
… find out information how to recognize if I am likely to be infected with the coronavirus?	☐	☐	☐	☐
… find information on how to find professional help in case of coronavirus infection?	☐	☐	☐	☐
… find information on how I much I am at risk for infection with coronavirus?	☐	☐	☐	☐
… understand your doctor`s, pharmacist`s or nurse`s instructions on protective measures against coronavirus infection?	☐	☐	☐	☐
… understand recommendations of authorities regarding protective measures against coronavirus infection?	☐	☐	☐	☐
… understand advice from family members or friends regarding protective measures against coronavirus infection?	☐	☐	☐	☐
… understand information in the media on how to protect myself against coronavirus infection?	☐	☐	☐	☐
… understand risks of the coronavirus that I find on the internet?	☐	☐	☐	☐
… understand risks of the coronavirus that I find in newspapers, magazines or on tv?	☐	☐	☐	☐
… judge if information on coronavirus and the coronavirus epidemic in the media is reliable?	☐	☐	☐	☐
… judge which behaviours are associated with higher risk of coronavirus infection?	☐	☐	☐	☐
… judge what protective measures you can apply to prevent a coronavirus infection?	☐	☐	☐	☐
… judge how much I am at risk for a coronavirus infection?	☐	☐	☐	☐
… judge if I have been infected with coronavirus?	☐	☐	☐	☐

	Not true at all	Mostly not true	Likely to be true	Totally true
… decide how you can protect yourself from coronavirus infection based on information in the media?	☐	☐	☐	☐
… follow instructions from your doctor or pharmacist regarding how to handle the coronavirus situation?	☐	☐	☐	☐
… use information the doctor gives you to decide how to handle an infection with coronavirus?	☐	☐	☐	☐
… use media information to decide how to handle an infection with coronavirus?	☐	☐	☐	☐
… to behave in a way to avoid infecting others?	☐	☐	☐	☐

In the following, we would like to know whether and to what extent you implement health promotion and prevention activities at your school.

25. Now we have a few questions about whether and to what extent health issues are addressed at your school in the context of the current COVID-19 pandemic, among other things.
At our school, …

	Not true at all	Mostly not true	Likely to be true	Totally true
…students are taught basic information about the coronavirus (e.g. causes of its development, spread).	☐	☐	☐	☐
…students learn ways to protect themselves from infection.	☐	☐	☐	☐
…students learn how to get enough exercise despite the restrictions due to the coronavirus.	☐	☐	☐	☐
…students learn how to eat healthily despite the restrictions due to the coronavirus.	☐	☐	☐	☐
…students are supported in dealing with worries and fears caused by the coronavirus.	☐	☐	☐	☐
…school staff are supported in dealing with stressful situations caused by the coronavirus (e.g. stress).	☐	☐	☐	☐
…health-promoting aspects play an important role in the design of teaching and learning conditions (including homeschooling).	☐	☐	☐	☐
…health promoting aspects play an important role in the design of working conditions (including home office).	☐	☐	☐	☐
…there are regular further training courses on health-related topics (protection against infection with the coronavirus, dealing with stressed students).	☐	☐	☐	☐
…stress resulting from the COVID-19 pandemic (workloads, student stress) are regularly addressed.	☐	☐	☐	☐
…we work closely with parents when it comes to promoting and protecting children's health.	☐	☐	☐	☐
…we work closely with community stakeholders from the health and social sectors when it comes to promoting and protecting the health of our students.	☐	☐	☐	☐
…there is a consensus that health and school performance of students are interrelated.	☐	☐	☐	☐

...students are involved in the planning of prevention and health promotion activities.	☐	☐	☐	☐
...(digital) spaces of social interaction and exchange are created despite the corona-related restrictions.	☐	☐	☐	☐

Finally, the following questions are about your health and the health of your students and teachers. We would like to know how you are doing.

26. **In the following, we would like to ask for your personal assessment of various aspects of student health at your school.**

Please rate how important you think the following health issues are for your students since the outbreak of the COVID-19 pandemic in [include the month] 2020.

	Very little importance	Little importance	High importance	Very high importance
Stress and coping	☐	☐	☐	☐
Internalised problems (e.g., anxierty, depression)	☐	☐	☐	☐
Behavioural problems (e.g., bullying)	☐	☐	☐	☐
Substance use (e.g., alcohol, tobacco)	☐	☐	☐	☐
Media use	☐	☐	☐	☐
Overweight	☐	☐	☐	☐
Healthy eating/nutrition	☐	☐	☐	☐
Physical activity/sport	☐	☐	☐	☐

27. **In the following, we would like to ask for your personal assessment of various aspects of teacher health at your school.**

Please rate how important you think the following health issues are for your teachers since the outbreak of the COVID-19 pandemic in [include the month] 2020.

	Very little importance	Little importance	High impirtance	Very high importance
Stress and coping	☐	☐	☐	☐
Internalised problems (e.g., anxierty, depression)	☐	☐	☐	☐
Anger management and impulse control	☐	☐	☐	☐
Substance use (e.g., alcohol, tobacco)	☐	☐	☐	☐
Media use	☐	☐	☐	☐
Overweight	☐	☐	☐	☐
Healthy eating/nutrition	☐	☐	☐	☐
Physical activity/sport	☐	☐	☐	☐

28. How is your health in general?

- ☐ Very good
- ☐ Good
- ☐ Moderate
- ☐ Bad
- ☐ Very bad

29. Do you have a chronic disease or a long-lasting health problem?
This refers to diseases or health problems that last or are expected to last at least 6 months.

☐ no ☐ yes

30. To what extent are you impaired by your chronic illness in activities of normal everyday life?

☐ Not at all impaired ☐ Moderately impaired ☐ Strongly impaired

31. The following statements concern your well-being during the last two weeks.
Please indicate for each of the five statements which is closest to how you have been feeling over the last two weeks.
Over the last two weeks …

	All of the time	Most of the time	More than half of the time	Less than half of the time	Some of the time	At no time
…I have felt cheerful and in good spirits	☐	☐	☐	☐	☐	☐
…I have felt calm and relaxed	☐	☐	☐	☐	☐	☐
…I have felt active and vigorous	☐	☐	☐	☐	☐	☐
…I woke up feeling fresh and rested	☐	☐	☐	☐	☐	☐
…my daily life has been filled with things that interest me	☐	☐	☐	☐	☐	☐

32. The following statements are related to your work situation and how you experience this situation.
Please state how often each statement applies to you.

	Never	Rarely	Sometimes	Often	Always
At work, I feel mentally exhausted.	☐	☐	☐	☐	☐
After a day at work, I find it hard to recover my energy.	☐	☐	☐	☐	☐
At work, I feel physically exhausted.	☐	☐	☐	☐	☐

33.	How often do you suffer from the following complaints?				
		Never	Rarely	Some-times	Often	Always

	Never	Rarely	Some-times	Often	Always
I suffer from palpitations or chest pain.	☐	☐	☐	☐	☐
I suffer from stomach and/or intestinal com-plaints.	☐	☐	☐	☐	☐
I suffer from headaches.	☐	☐	☐	☐	☐
I suffer from muscle pain, for example in the neck, shoulder or back.	☐	☐	☐	☐	☐
I often get sick.	☐	☐	☐	☐	☐

You made it! Thank you very much for your participation.

The results of this study are published on the websites of the universities responsible for the study [include your universities here].

You can now close the page.
