# Supplementary material for: Associations between Polish school principals’ health literacy and implementation of the Health Promoting School approach during the COVID-19 pandemic
Source: PLoS One. 2024 Apr 2;19(4):e0301055. doi: 10.1371/journal.pone.0301055 (PMC10986982; doi:10.1371/journal.pone.0301055)
Supplement: S2 Appendix — (ZIP) [file pone.0301055.s002.zip › HPS descripive statistics.pdf]

Whether and to what extent health issues are addressed at your school in the context of the current COVID-19 pandemic, among other things. At our school, …

Students are taught basic information about the coronavirus (e.g. causes of its development, spread)					
		Frequency	Percentage	Valid percentage	Cumulative percentage
Valid	Not true at all	27	1,4	3,2	3,2
	Mostly not true	6	0,3	0,7	3,9
	Likely to be true	115	6,1	13,5	17,4
	Totally true	705	37,1	82,6	100,0
	Total	853	44,9	100,0	
Missing data		1046	55,1		
Total		1899	100,0		

Students learn ways to protect themselves from infection					
		Frequency	Percentage	Valid percentage	Cumulative percentage
Valid	Not true at all	28	1,5	3,3	3,3
	Mostly not true	5	0,3	0,6	3,9
	Likely to be true	156	8,2	18,3	22,2
	Totally true	664	35,0	77,8	100,0
	Total	853	44,9	100,0	
Missing data		1046	55,1		
Total		1899	100,0		

Students learn how to get enough exercise despite the restrictions due to the coronavirus					
		Frequency	Percentage	Valid percentage	Cumulative percentage
Valid	Not true at all	28	1,5	3,3	3,3
	Mostly not true	10	0,5	1,2	4,5
	Likely to be true	248	13,1	29,1	33,6
	Totally true	566	29,8	66,4	100,0
	Total	852	44,9	100,0	
Missing data		1047	55,1		
Total		1899	100,0		

Students learn how to eat healthily despite the restrictions due to the coronavirus					
		Frequency	Percentage	Valid percentage	Cumulative percentage
Valid	Not true at all	28	1,5	3,3	3,3
	Mostly not true	19	1,0	2,2	5,5
	Likely to be true	269	14,2	31,5	37,0
	Totally true	537	28,3	63,0	100,0
	Total	853	44,9	100,0	
Missing data		1046	55,1		
Total		1899	100,0		

Students are supported in dealing with worries and fears caused by the coronavirus					
		Frequency	Percentage	Valid percentage	Cumulative percentage
Valid	Not true at all	27	1,4	3,2	3,2
	Mostly not true	15	0,8	1,8	4,9
	Likely to be true	271	14,3	31,8	36,7
	Totally true	540	28,4	63,3	100,0
	Total	853	44,9	100,0	
Missing data		1046	55,1		
Total		1899	100,0		

School staff are supported in dealing with stressful situations caused by the coronavirus (e.g. stress)					
		Frequency	Percentage	Valid percentage	Cumulative percentage
Valid	Not true at all	28	1,5	3,3	3,3
	Mostly not true	54	2,8	6,3	9,6
	Likely to be true	330	17,4	38,7	48,3
	Totally true	441	23,2	51,7	100,0
	Total	853	44,9	100,0	
Missing data		1046	55,1		
Total		1899	100,0		

Health-promoting aspects play an important role in the design of teaching and learning conditions (including homeschooling)					
		Frequency	Percentage	Valid percentage	Cumulative percentage
Valid	Not true at all	27	1,4	3,2	3,2
	Mostly not true	26	1,4	3,1	6,2
	Likely to be true	325	17,1	38,1	44,4
	Totally true	474	25,0	55,6	100,0
	Total	852	44,9	100,0	
Missing data		1047	55,1		
Total		1899	100,0		

There are regular further training courses on health-related topics (protection against infection with the coronavirus, dealing with stressed students)					
		Frequency	Percentage	Valid percentage	Cumulative percentage
Valid	Not true at all	30	1,6	3,5	3,5
	Mostly not true	173	9,1	20,3	23,8
	Likely to be true	409	21,5	48,0	71,8
	Totally true	240	12,6	28,2	100,0
	Total	852	44,9	100,0	
Missing data		1047	55,1		
Total		1899	100,0		

Stress resulting from the COVID-19 pandemic (workloads, student stress) are regularly addressed					
		Frequency	Percentage	Valid percentage	Cumulative percentage
Valid	Not true at all	27	1,4	3,2	3,2
	Mostly not true	101	5,3	11,9	15,0
	Likely to be true	433	22,8	50,8	65,8
	Totally true	291	15,3	34,2	100,0
	Total	852	44,9	100,0	
Missing data		1047	55,1		
Total		1899	100,0		

We work closely with parents when it comes to promoting and protecting children's health					
		Frequency	Percentage	Valid percentage	Cumulative percentage
Valid	Not true at all	28	1,5	3,3	3,3
	Mostly not true	26	1,4	3,1	6,3
	Likely to be true	370	19,5	43,5	49,8
	Totally true	427	22,5	50,2	100,0
	Total	851	44,8	100,0	
Missing data		1048	55,2		
Total		1899	100,0		

We work closely with community stakeholders from the health and social sectors when it comes to promoting and protecting the health of our students					
		Frequency	Percentage	Valid percentage	Cumulative percentage
Valid	Not true at all	30	1,6	3,5	3,5
	Mostly not true	103	5,4	12,1	15,7
	Likely to be true	410	21,6	48,3	64,0
	Totally true	306	16,1	36,0	100,0
	Total	849	44,7	100,0	
Missing data		1050	55,3		
Total		1899	100,0		

There is a consensus that health and school performance of students are interrelated					
		Frequency	Percentage	Valid percentage	Cumulative percentage
Valid	Not true at all	25	1,3	2,9	2,9
	Mostly not true	30	1,6	3,5	6,5
	Likely to be true	393	20,7	46,1	52,6
	Totally true	404	21,3	47,4	100,0
	Total	852	44,9	100,0	
Missing data		1047	55,1		
Total		1899	100,0		

Students are involved in the planning of prevention and health promotion activities					
		Frequency	Percentage	Valid percentage	Cumulative percentage
Valid	Not true at all	24	1,3	2,8	2,8
	Mostly not true	73	3,8	8,6	11,4
	Likely to be true	426	22,4	49,9	61,3
	Totally true	330	17,4	38,7	100,0
	Total	853	44,9	100,0	
Missing data		1046	55,1		
Total		1899	100,0		

(Digital) spaces of social interaction and exchange are created despite the corona-related restrictions					
		Frequency	Percentage	Valid percentage	Cumulative percentage
Valid	Not true at all	28	1,5	3,3	3,3
	Mostly not true	58	3,1	6,8	10,1
	Likely to be true	407	21,4	47,9	58,0
	Totally true	357	18,8	42,0	100,0
	Total	850	44,8	100,0	
Missing data		1049	55,2		
Total		1899	100,0		
