# Supplementary material for: Associations between Polish school principals’ health literacy and implementation of the Health Promoting School approach during the COVID-19 pandemic
Source: PLoS One. 2024 Apr 2;19(4):e0301055. doi: 10.1371/journal.pone.0301055 (PMC10986982; doi:10.1371/journal.pone.0301055)
Supplement: S2 Appendix — (ZIP) [file pone.0301055.s002.zip › School principals HL - descriptive statistics.pdf]

On a scale from very easy to very difficult, how easy would you say it is to…

find information about the coronavirus on the internet?					
		Frequency	Percentage	Valid percentage	Cumulative percentage
Valid	Not true at all	1	0,1	0,1	0,1
	Mostly not true	5	0,3	0,6	0,7
	Likely to be true	294	15,5	33,5	34,2
	Totally true	577	30,4	65,8	100,0
	Total	877	46,2	100,0	
Missing data		1022	53,8		
Total		1899	100,0		

find information on the internet about protective behaviours that can help to prevent infection with the coronavirus?					
		Frequency	Percentage	Valid percentage	Cumulative percentage
Valid	Not true at all	0	0,0	0,0	0,0
	Mostly not true	11	0,6	1,3	1,3
	Likely to be true	315	16,6	36,3	37,6
	Totally true	542	28,5	62,4	100,0
	Total	868	45,7	100,0	
Missing data		1031	54,3		
Total		1899	100,0		

find information in newspapers, magazines and on tv about behaviours that can help to prevent infection with the coronavirus?					
		Frequency	Percentage	Valid percentage	Cumulative percentage
Valid	Not true at all	5	0,3	0,6	0,6
	Mostly not true	42	2,2	4,8	5,4
	Likely to be true	394	20,7	45,1	50,5
	Totally true	433	22,8	49,5	100,0
	Total	874	46,0	100,0	
Missing data		1025	54,0		
Total		1899	100,0		

find out information how to recognize if I am likely to be infected with the coronavirus?					
		Frequency	Percentage	Valid percentage	Cumulative percentage
Valid	Not true at all	1	0,1	0,1	0,1
	Mostly not true	50	2,6	5,7	5,8
	Likely to be true	434	22,9	49,6	55,4
	Totally true	390	20,5	44,6	100,0
	Total	875	46,1	100,0	
Missing data		1024	53,9		
Total		1899	100,0		

find information on how to find professional help in case of coronavirus infection?					
		Frequency	Percentage	Valid percentage	Cumulative percentage
Valid	Not true at all	15	0,8	1,7	1,7
	Mostly not true	127	6,7	14,5	16,3
	Likely to be true	415	21,9	47,5	63,8
	Totally true	316	16,6	36,2	100,0
	Total	873	46,0	100,0	
Missing data		1026	54,0		
Total		1899	100,0		

find information on how I much I am at risk for infection with coronavirus?					
		Frequency	Percentage	Valid percentage	Cumulative percentage
Valid	Not true at all	12	0,6	1,4	1,4
	Mostly not true	110	5,8	12,6	14,0
	Likely to be true	440	23,2	50,5	64,5
	Totally true	309	16,3	35,5	100,0
	Total	871	45,9	100,0	
Missing data		1028	54,1		
Total		1899	100,0		

understand your doctor`s, pharmacist`s or nurse`s instructions on protective measures against coronavirus infection?					
		Frequency	Percentage	Valid percentage	Cumulative percentage
Valid	Not true at all	19	1,0	2,2	2,2
	Mostly not true	83	4,4	9,5	11,7
	Likely to be true	436	23,0	49,9	61,6
	Totally true	335	17,6	38,4	100,0
	Total	873	46,0	100,0	
Missing data		1026	54,0		
Total		1899	100,0		

understand recommendations of authorities regarding protective measures against coronavirus infection?					
		Frequency	Percentage	Valid percentage	Cumulative percentage
Valid	Not true at all	62	3,3	7,1	7,1
	Mostly not true	181	9,5	20,7	27,8
	Likely to be true	375	19,7	43,0	70,8
	Totally true	255	13,4	29,2	100,0
	Total	873	46,0	100,0	
Missing data		1026	54,0		
Total		1899	100,0		

understand advice from family members or friends regarding protective measures against coronavirus infection?					
		Frequency	Percentage	Valid percentage	Cumulative percentage
Valid	Not true at all	7	0,4	0,8	0,8
	Mostly not true	84	4,4	9,6	10,4
	Likely to be true	486	25,6	55,7	66,2
	Totally true	295	15,5	33,8	100,0
	Total	872	45,9	100,0	
Missing data		1027	54,1		
Total		1899	100,0		

understand information in the media on how to protect myself against coronavirus infection?					
		Frequency	Percentage	Valid percentage	Cumulative percentage
Valid	Not true at all	10	0,5	1,1	1,1
	Mostly not true	60	3,2	6,9	8,0
	Likely to be true	458	24,1	52,6	60,7
	Totally true	342	18,0	39,3	100,0
	Total	870	45,8	100,0	
Missing data		1029	54,2		
Total		1899	100,0		

understand risks of the coronavirus that I find on the internet?					
		Frequency	Percentage	Valid percentage	Cumulative percentage
Valid	Not true at all	7	0,4	0,8	0,8
	Mostly not true	57	3,0	6,5	7,3
	Likely to be true	452	23,8	51,9	59,2
	Totally true	355	18,7	40,8	100,0
	Total	871	45,9	100,0	
Missing data		1028	54,1		
Total		1899	100,0		

understand risks of the coronavirus that I find in newspapers, magazines or on tv?					
		Frequency	Percentage	Valid percentage	Cumulative percentage
Valid	Not true at all	14	0,7	1,6	1,6
	Mostly not true	78	4,1	9,0	10,6
	Likely to be true	461	24,3	53,0	63,6
	Totally true	317	16,7	36,4	100,0
	Total	870	45,8	100,0	
Missing data		1029	54,2		
Total		1899	100,0		

judge if information on coronavirus and the coronavirus epidemic in the media is reliable?					
		Frequency	Percentage	Valid percentage	Cumulative percentage
Valid	Not true at all	56	2,9	6,4	6,4
	Mostly not true	281	14,8	32,3	38,8
	Likely to be true	357	18,8	41,1	79,9
	Totally true	175	9,2	20,1	100,0
	Total	869	45,8	100,0	
Missing data		1030	54,2		
Total		1899	100,0		

judge which behaviours are associated with higher risk of coronavirus infection?					
		Frequency	Percentage	Valid percentage	Cumulative percentage
Valid	Not true at all	18	0,9	2,1	2,1
	Mostly not true	136	7,2	15,7	17,8
	Likely to be true	451	23,7	52,1	69,9
	Totally true	261	13,7	30,1	100,0
	Total	866	45,6	100,0	
Missing data		1033	54,4		
Total		1899	100,0		

judge what protective measures you can apply to prevent a coronavirus infection?					
		Frequency	Percentage	Valid percentage	Cumulative percentage
Valid	Not true at all	5	0,3	0,6	0,6
	Mostly not true	80	4,2	9,2	9,8
	Likely to be true	473	24,9	54,3	64,1
	Totally true	313	16,5	35,9	100,0
	Total	871	45,9	100,0	
Missing data		1028	54,1		
Total		1899	100,0		

judge how much I am at risk for a coronavirus infection?					
		Frequency	Percentage	Valid percentage	Cumulative percentage
Valid	Not true at all	19	1,0	2,2	2,2
	Mostly not true	188	9,9	21,7	23,8
	Likely to be true	430	22,6	49,5	73,4
	Totally true	231	12,2	26,6	100,0
	Total	868	45,7	100,0	
Missing data		1031	54,3		
Total		1899	100,0		

judge if I have been infected with coronavirus?					
		Frequency	Percentage	Valid percentage	Cumulative percentage
Valid	Not true at all	33	1,7	3,8	3,8
	Mostly not true	273	14,4	31,5	35,3
	Likely to be true	383	20,2	44,2	79,6
	Totally true	177	9,3	20,4	100,0
	Total	866	45,6	100,0	
Missing data		1033	54,4		
Total		1899	100,0		

decide how you can protect yourself from coronavirus infection based on information in the media?					
		Frequency	Percentage	Valid percentage	Cumulative percentage
Valid	Not true at all	8	0,4	0,9	0,9
	Mostly not true	97	5,1	11,1	12,1
	Likely to be true	487	25,6	55,9	68,0
	Totally true	279	14,7	32,0	100,0
	Total	871	45,9	100,0	
Missing data		1028	54,1		
Total		1899	100,0		

follow instructions from your doctor or pharmacist regarding how to handle the coronavirus situation?					
		Frequency	Percentage	Valid percentage	Cumulative percentage
Valid	Not true at all	13	0,7	1,5	1,5
	Mostly not true	78	4,1	9,0	10,5
	Likely to be true	480	25,3	55,4	65,9
	Totally true	296	15,6	34,1	100,0
	Total	867	45,7	100,0	
Missing data		1032	54,3		
Total		1899	100,0		

use information the doctor gives you to decide how to handle an infection with coronavirus?					
		Frequency	Percentage	Valid percentage	Cumulative percentage
Valid	Not true at all	13	0,7	1,5	1,5
	Mostly not true	54	2,8	6,2	7,7
	Likely to be true	497	26,2	57,1	64,8
	Totally true	307	16,2	35,2	100,0
	Total	871	45,9	100,0	
Missing data		1028	54,1		
Total		1899	100,0		

use media information to decide how to handle an infection with coronavirus?					
		Frequency	Percentage	Valid percentage	Cumulative percentage
Valid	Not true at all	15	0,8	1,7	1,7
	Mostly not true	75	3,9	8,6	10,3
	Likely to be true	504	26,5	57,9	68,2
	Totally true	277	14,6	31,8	100,0
	Total	871	45,9	100,0	
Missing data		1028	54,1		
Total		1899	100,0		

to behave in a way to avoid infecting others?					
		Frequency	Percentage	Valid percentage	Cumulative percentage
Valid	Not true at all	12	0,6	1,4	1,4
	Mostly not true	92	4,8	10,6	12,0
	Likely to be true	458	24,1	52,6	64,6
	Totally true	308	16,2	35,4	100,0
	Total	870	45,8	100,0	
Missing data		1029	54,2		
Total		1899	100,0		
