# Supplementary material for: Associations between Polish school principals’ health literacy and implementation of the Health Promoting School approach during the COVID-19 pandemic
Source: PLoS One. 2024 Apr 2;19(4):e0301055. doi: 10.1371/journal.pone.0301055 (PMC10986982; doi:10.1371/journal.pone.0301055)
Supplement: S2 Appendix — (ZIP) [file pone.0301055.s002.zip › School principals survey - sample size.pdf]

Gender					
		Frequency	Percentage	Valid percentage	Cumulative percentage
Valid	Men	198	10,4	18,0	18,0
	Female	901	47,4	82,0	100,0
	Total	1099	57,9	100,0	
Missing data		800	42,1		
Total		1899	100,0		

Age range					
		Frequency	Percentage	Valid percentage	Cumulative percentage
Valid	30-44	158	8,3	14,5	14,5
	45-54	499	26,3	45,9	60,4
	>55	430	22,6	39,6	100,0
	Total	1087	57,2	100,0	
Missing data		812	42,8		
Total		1899	100,0		

Type of school					
		Frequency	Percentage	Valid percentage	Cumulative percentage
Valid	primary school	810	42,7	78,6	78,6
	secondary school	220	11,6	21,4	100,0
	Total	1030	54,2	100,0	
Missing data		869	45,8		
Total		1899	100,0		

Respondent position					
		Frequency	Percentage	Valid percentage	Cumulative percentage
Valid	School principal	810	42,7	74,2	74,2
	Vice-principal	281	14,8	25,8	100,0
	Total	1091	57,5	100,0	
Missing data		808	42,5		
Total		1899	100,0		
