# Supplementary material for: Associations between Polish school principals’ health literacy and implementation of the Health Promoting School approach during the COVID-19 pandemic
Source: PLoS One. 2024 Apr 2;19(4):e0301055. doi: 10.1371/journal.pone.0301055 (PMC10986982; doi:10.1371/journal.pone.0301055)
Supplement: S2 Appendix — (ZIP) [file pone.0301055.s002.zip › HPS descripive statistics.docx]

**Whether and to what extent health issues are addressed at your school in the context of the current COVID-19 pandemic, among other things. At our school, …**

| Students are taught basic information about the coronavirus (e.g. causes of its development, spread) | | | | | |
| --- | --- | --- | --- | --- | --- |
|  | | Frequency | Percentage | Valid percentage | Cumulative percentage |
| Valid | Not true at all | 27 | 1,4 | 3,2 | 3,2 |
|  | Mostly not true | 6 | 0,3 | 0,7 | 3,9 |
|  | Likely to be true | 115 | 6,1 | 13,5 | 17,4 |
|  | Totally true | 705 | 37,1 | 82,6 | 100,0 |
|  | Total | 853 | 44,9 | 100,0 |  |
| Missing data | | 1046 | 55,1 |  |  |
| Total | | 1899 | 100,0 |  |  |

| Students learn ways to protect themselves from infection | | | | | |
| --- | --- | --- | --- | --- | --- |
|  | | Frequency | Percentage | Valid percentage | Cumulative percentage |
| Valid | Not true at all | 28 | 1,5 | 3,3 | 3,3 |
|  | Mostly not true | 5 | 0,3 | 0,6 | 3,9 |
|  | Likely to be true | 156 | 8,2 | 18,3 | 22,2 |
|  | Totally true | 664 | 35,0 | 77,8 | 100,0 |
|  | Total | 853 | 44,9 | 100,0 |  |
| Missing data | | 1046 | 55,1 |  |  |
| Total | | 1899 | 100,0 |  |  |

| Students learn how to get enough exercise despite the restrictions due to the coronavirus | | | | | |
| --- | --- | --- | --- | --- | --- |
|  | | Frequency | Percentage | Valid percentage | Cumulative percentage |
| Valid | Not true at all | 28 | 1,5 | 3,3 | 3,3 |
|  | Mostly not true | 10 | 0,5 | 1,2 | 4,5 |
|  | Likely to be true | 248 | 13,1 | 29,1 | 33,6 |
|  | Totally true | 566 | 29,8 | 66,4 | 100,0 |
|  | Total | 852 | 44,9 | 100,0 |  |
| Missing data | | 1047 | 55,1 |  |  |
| Total | | 1899 | 100,0 |  |  |

| Students learn how to eat healthily despite the restrictions due to the coronavirus | | | | | |
| --- | --- | --- | --- | --- | --- |
|  | | Frequency | Percentage | Valid percentage | Cumulative percentage |
| Valid | Not true at all | 28 | 1,5 | 3,3 | 3,3 |
|  | Mostly not true | 19 | 1,0 | 2,2 | 5,5 |
|  | Likely to be true | 269 | 14,2 | 31,5 | 37,0 |
|  | Totally true | 537 | 28,3 | 63,0 | 100,0 |
|  | Total | 853 | 44,9 | 100,0 |  |
| Missing data | | 1046 | 55,1 |  |  |
| Total | | 1899 | 100,0 |  |  |

| Students are supported in dealing with worries and fears caused by the coronavirus | | | | | |
| --- | --- | --- | --- | --- | --- |
|  | | Frequency | Percentage | Valid percentage | Cumulative percentage |
| Valid | Not true at all | 27 | 1,4 | 3,2 | 3,2 |
|  | Mostly not true | 15 | 0,8 | 1,8 | 4,9 |
|  | Likely to be true | 271 | 14,3 | 31,8 | 36,7 |
|  | Totally true | 540 | 28,4 | 63,3 | 100,0 |
|  | Total | 853 | 44,9 | 100,0 |  |
| Missing data | | 1046 | 55,1 |  |  |
| Total | | 1899 | 100,0 |  |  |

| School staff are supported in dealing with stressful situations caused by the coronavirus (e.g. stress) | | | | | |
| --- | --- | --- | --- | --- | --- |
|  | | Frequency | Percentage | Valid percentage | Cumulative percentage |
| Valid | Not true at all | 28 | 1,5 | 3,3 | 3,3 |
|  | Mostly not true | 54 | 2,8 | 6,3 | 9,6 |
|  | Likely to be true | 330 | 17,4 | 38,7 | 48,3 |
|  | Totally true | 441 | 23,2 | 51,7 | 100,0 |
|  | Total | 853 | 44,9 | 100,0 |  |
| Missing data | | 1046 | 55,1 |  |  |
| Total | | 1899 | 100,0 |  |  |

| Health-promoting aspects play an important role in the design of teaching and learning conditions (including homeschooling) | | | | | |
| --- | --- | --- | --- | --- | --- |
|  | | Frequency | Percentage | Valid percentage | Cumulative percentage |
| Valid | Not true at all | 27 | 1,4 | 3,2 | 3,2 |
|  | Mostly not true | 26 | 1,4 | 3,1 | 6,2 |
|  | Likely to be true | 325 | 17,1 | 38,1 | 44,4 |
|  | Totally true | 474 | 25,0 | 55,6 | 100,0 |
|  | Total | 852 | 44,9 | 100,0 |  |
| Missing data | | 1047 | 55,1 |  |  |
| Total | | 1899 | 100,0 |  |  |

| There are regular further training courses on health-related topics (protection against infection with the coronavirus, dealing with stressed students) | | | | | |
| --- | --- | --- | --- | --- | --- |
|  | | Frequency | Percentage | Valid percentage | Cumulative percentage |
| Valid | Not true at all | 30 | 1,6 | 3,5 | 3,5 |
|  | Mostly not true | 173 | 9,1 | 20,3 | 23,8 |
|  | Likely to be true | 409 | 21,5 | 48,0 | 71,8 |
|  | Totally true | 240 | 12,6 | 28,2 | 100,0 |
|  | Total | 852 | 44,9 | 100,0 |  |
| Missing data | | 1047 | 55,1 |  |  |
| Total | | 1899 | 100,0 |  |  |

| Stress resulting from the COVID-19 pandemic (workloads, student stress) are regularly addressed | | | | | |
| --- | --- | --- | --- | --- | --- |
|  | | Frequency | Percentage | Valid percentage | Cumulative percentage |
| Valid | Not true at all | 27 | 1,4 | 3,2 | 3,2 |
|  | Mostly not true | 101 | 5,3 | 11,9 | 15,0 |
|  | Likely to be true | 433 | 22,8 | 50,8 | 65,8 |
|  | Totally true | 291 | 15,3 | 34,2 | 100,0 |
|  | Total | 852 | 44,9 | 100,0 |  |
| Missing data | | 1047 | 55,1 |  |  |
| Total | | 1899 | 100,0 |  |  |

| We work closely with parents when it comes to promoting and protecting children's health | | | | | |
| --- | --- | --- | --- | --- | --- |
|  | | Frequency | Percentage | Valid percentage | Cumulative percentage |
| Valid | Not true at all | 28 | 1,5 | 3,3 | 3,3 |
|  | Mostly not true | 26 | 1,4 | 3,1 | 6,3 |
|  | Likely to be true | 370 | 19,5 | 43,5 | 49,8 |
|  | Totally true | 427 | 22,5 | 50,2 | 100,0 |
|  | Total | 851 | 44,8 | 100,0 |  |
| Missing data | | 1048 | 55,2 |  |  |
| Total | | 1899 | 100,0 |  |  |

| We work closely with community stakeholders from the health and social sectors when it comes to promoting and protecting the health of our students | | | | | |
| --- | --- | --- | --- | --- | --- |
|  | | Frequency | Percentage | Valid percentage | Cumulative percentage |
| Valid | Not true at all | 30 | 1,6 | 3,5 | 3,5 |
|  | Mostly not true | 103 | 5,4 | 12,1 | 15,7 |
|  | Likely to be true | 410 | 21,6 | 48,3 | 64,0 |
|  | Totally true | 306 | 16,1 | 36,0 | 100,0 |
|  | Total | 849 | 44,7 | 100,0 |  |
| Missing data | | 1050 | 55,3 |  |  |
| Total | | 1899 | 100,0 |  |  |

| There is a consensus that health and school performance of students are interrelated | | | | | |
| --- | --- | --- | --- | --- | --- |
|  | | Frequency | Percentage | Valid percentage | Cumulative percentage |
| Valid | Not true at all | 25 | 1,3 | 2,9 | 2,9 |
|  | Mostly not true | 30 | 1,6 | 3,5 | 6,5 |
|  | Likely to be true | 393 | 20,7 | 46,1 | 52,6 |
|  | Totally true | 404 | 21,3 | 47,4 | 100,0 |
|  | Total | 852 | 44,9 | 100,0 |  |
| Missing data | | 1047 | 55,1 |  |  |
| Total | | 1899 | 100,0 |  |  |

| Students are involved in the planning of prevention and health promotion activities | | | | | |
| --- | --- | --- | --- | --- | --- |
|  | | Frequency | Percentage | Valid percentage | Cumulative percentage |
| Valid | Not true at all | 24 | 1,3 | 2,8 | 2,8 |
|  | Mostly not true | 73 | 3,8 | 8,6 | 11,4 |
|  | Likely to be true | 426 | 22,4 | 49,9 | 61,3 |
|  | Totally true | 330 | 17,4 | 38,7 | 100,0 |
|  | Total | 853 | 44,9 | 100,0 |  |
| Missing data | | 1046 | 55,1 |  |  |
| Total | | 1899 | 100,0 |  |  |

| (Digital) spaces of social interaction and exchange are created despite the corona-related restrictions | | | | | |
| --- | --- | --- | --- | --- | --- |
|  | | Frequency | Percentage | Valid percentage | Cumulative percentage |
| Valid | Not true at all | 28 | 1,5 | 3,3 | 3,3 |
|  | Mostly not true | 58 | 3,1 | 6,8 | 10,1 |
|  | Likely to be true | 407 | 21,4 | 47,9 | 58,0 |
|  | Totally true | 357 | 18,8 | 42,0 | 100,0 |
|  | Total | 850 | 44,8 | 100,0 |  |
| Missing data | | 1049 | 55,2 |  |  |
| Total | | 1899 | 100,0 |  |  |
