# Supplementary material for: Associations between Polish school principals’ health literacy and implementation of the Health Promoting School approach during the COVID-19 pandemic
Source: PLoS One. 2024 Apr 2;19(4):e0301055. doi: 10.1371/journal.pone.0301055 (PMC10986982; doi:10.1371/journal.pone.0301055)
Supplement: S2 Appendix — (ZIP) [file pone.0301055.s002.zip › School principals HL - descriptive statistics.docx]

**On a scale from very easy to very difficult, how easy would you say it is to…**

| find information about the coronavirus on the internet? | | | | | |
| --- | --- | --- | --- | --- | --- |
|  | | Frequency | Percentage | Valid percentage | Cumulative percentage |
| Valid | Not true at all | 1 | 0,1 | 0,1 | 0,1 |
|  | Mostly not true | 5 | 0,3 | 0,6 | 0,7 |
|  | Likely to be true | 294 | 15,5 | 33,5 | 34,2 |
|  | Totally true | 577 | 30,4 | 65,8 | 100,0 |
|  | Total | 877 | 46,2 | 100,0 |  |
| Missing data | | 1022 | 53,8 |  |  |
| Total | | 1899 | 100,0 |  |  |

| find information on the internet about protective behaviours that can help to prevent infection with the coronavirus? | | | | | |
| --- | --- | --- | --- | --- | --- |
|  | | Frequency | Percentage | Valid percentage | Cumulative percentage |
| Valid | Not true at all | 0 | 0,0 | 0,0 | 0,0 |
|  | Mostly not true | 11 | 0,6 | 1,3 | 1,3 |
|  | Likely to be true | 315 | 16,6 | 36,3 | 37,6 |
|  | Totally true | 542 | 28,5 | 62,4 | 100,0 |
|  | Total | 868 | 45,7 | 100,0 |  |
| Missing data | | 1031 | 54,3 |  |  |
| Total | | 1899 | 100,0 |  |  |

| find information in newspapers, magazines and on tv about behaviours that can help to prevent infection with the coronavirus? | | | | | |
| --- | --- | --- | --- | --- | --- |
|  | | Frequency | Percentage | Valid percentage | Cumulative percentage |
| Valid | Not true at all | 5 | 0,3 | 0,6 | 0,6 |
|  | Mostly not true | 42 | 2,2 | 4,8 | 5,4 |
|  | Likely to be true | 394 | 20,7 | 45,1 | 50,5 |
|  | Totally true | 433 | 22,8 | 49,5 | 100,0 |
|  | Total | 874 | 46,0 | 100,0 |  |
| Missing data | | 1025 | 54,0 |  |  |
| Total | | 1899 | 100,0 |  |  |

| find out information how to recognize if I am likely to be infected with the coronavirus? | | | | | |
| --- | --- | --- | --- | --- | --- |
|  | | Frequency | Percentage | Valid percentage | Cumulative percentage |
| Valid | Not true at all | 1 | 0,1 | 0,1 | 0,1 |
|  | Mostly not true | 50 | 2,6 | 5,7 | 5,8 |
|  | Likely to be true | 434 | 22,9 | 49,6 | 55,4 |
|  | Totally true | 390 | 20,5 | 44,6 | 100,0 |
|  | Total | 875 | 46,1 | 100,0 |  |
| Missing data | | 1024 | 53,9 |  |  |
| Total | | 1899 | 100,0 |  |  |

| find information on how to find professional help in case of coronavirus infection? | | | | | |
| --- | --- | --- | --- | --- | --- |
|  | | Frequency | Percentage | Valid percentage | Cumulative percentage |
| Valid | Not true at all | 15 | 0,8 | 1,7 | 1,7 |
|  | Mostly not true | 127 | 6,7 | 14,5 | 16,3 |
|  | Likely to be true | 415 | 21,9 | 47,5 | 63,8 |
|  | Totally true | 316 | 16,6 | 36,2 | 100,0 |
|  | Total | 873 | 46,0 | 100,0 |  |
| Missing data | | 1026 | 54,0 |  |  |
| Total | | 1899 | 100,0 |  |  |

| find information on how I much I am at risk for infection with coronavirus? | | | | | |
| --- | --- | --- | --- | --- | --- |
|  | | Frequency | Percentage | Valid percentage | Cumulative percentage |
| Valid | Not true at all | 12 | 0,6 | 1,4 | 1,4 |
|  | Mostly not true | 110 | 5,8 | 12,6 | 14,0 |
|  | Likely to be true | 440 | 23,2 | 50,5 | 64,5 |
|  | Totally true | 309 | 16,3 | 35,5 | 100,0 |
|  | Total | 871 | 45,9 | 100,0 |  |
| Missing data | | 1028 | 54,1 |  |  |
| Total | | 1899 | 100,0 |  |  |

| understand your doctor`s, pharmacist`s or nurse`s instructions on protective measures against coronavirus infection? | | | | | |
| --- | --- | --- | --- | --- | --- |
|  | | Frequency | Percentage | Valid percentage | Cumulative percentage |
| Valid | Not true at all | 19 | 1,0 | 2,2 | 2,2 |
|  | Mostly not true | 83 | 4,4 | 9,5 | 11,7 |
|  | Likely to be true | 436 | 23,0 | 49,9 | 61,6 |
|  | Totally true | 335 | 17,6 | 38,4 | 100,0 |
|  | Total | 873 | 46,0 | 100,0 |  |
| Missing data | | 1026 | 54,0 |  |  |
| Total | | 1899 | 100,0 |  |  |

| understand recommendations of authorities regarding protective measures against coronavirus infection? | | | | | |
| --- | --- | --- | --- | --- | --- |
|  | | Frequency | Percentage | Valid percentage | Cumulative percentage |
| Valid | Not true at all | 62 | 3,3 | 7,1 | 7,1 |
|  | Mostly not true | 181 | 9,5 | 20,7 | 27,8 |
|  | Likely to be true | 375 | 19,7 | 43,0 | 70,8 |
|  | Totally true | 255 | 13,4 | 29,2 | 100,0 |
|  | Total | 873 | 46,0 | 100,0 |  |
| Missing data | | 1026 | 54,0 |  |  |
| Total | | 1899 | 100,0 |  |  |

| understand advice from family members or friends regarding protective measures against coronavirus infection? | | | | | |
| --- | --- | --- | --- | --- | --- |
|  | | Frequency | Percentage | Valid percentage | Cumulative percentage |
| Valid | Not true at all | 7 | 0,4 | 0,8 | 0,8 |
|  | Mostly not true | 84 | 4,4 | 9,6 | 10,4 |
|  | Likely to be true | 486 | 25,6 | 55,7 | 66,2 |
|  | Totally true | 295 | 15,5 | 33,8 | 100,0 |
|  | Total | 872 | 45,9 | 100,0 |  |
| Missing data | | 1027 | 54,1 |  |  |
| Total | | 1899 | 100,0 |  |  |

| understand information in the media on how to protect myself against coronavirus infection? | | | | | |
| --- | --- | --- | --- | --- | --- |
|  | | Frequency | Percentage | Valid percentage | Cumulative percentage |
| Valid | Not true at all | 10 | 0,5 | 1,1 | 1,1 |
|  | Mostly not true | 60 | 3,2 | 6,9 | 8,0 |
|  | Likely to be true | 458 | 24,1 | 52,6 | 60,7 |
|  | Totally true | 342 | 18,0 | 39,3 | 100,0 |
|  | Total | 870 | 45,8 | 100,0 |  |
| Missing data | | 1029 | 54,2 |  |  |
| Total | | 1899 | 100,0 |  |  |

| understand risks of the coronavirus that I find on the internet? | | | | | |
| --- | --- | --- | --- | --- | --- |
|  | | Frequency | Percentage | Valid percentage | Cumulative percentage |
| Valid | Not true at all | 7 | 0,4 | 0,8 | 0,8 |
|  | Mostly not true | 57 | 3,0 | 6,5 | 7,3 |
|  | Likely to be true | 452 | 23,8 | 51,9 | 59,2 |
|  | Totally true | 355 | 18,7 | 40,8 | 100,0 |
|  | Total | 871 | 45,9 | 100,0 |  |
| Missing data | | 1028 | 54,1 |  |  |
| Total | | 1899 | 100,0 |  |  |

| understand risks of the coronavirus that I find in newspapers, magazines or on tv? | | | | | |
| --- | --- | --- | --- | --- | --- |
|  | | Frequency | Percentage | Valid percentage | Cumulative percentage |
| Valid | Not true at all | 14 | 0,7 | 1,6 | 1,6 |
|  | Mostly not true | 78 | 4,1 | 9,0 | 10,6 |
|  | Likely to be true | 461 | 24,3 | 53,0 | 63,6 |
|  | Totally true | 317 | 16,7 | 36,4 | 100,0 |
|  | Total | 870 | 45,8 | 100,0 |  |
| Missing data | | 1029 | 54,2 |  |  |
| Total | | 1899 | 100,0 |  |  |

| judge if information on coronavirus and the coronavirus epidemic in the media is reliable? | | | | | |
| --- | --- | --- | --- | --- | --- |
|  | | Frequency | Percentage | Valid percentage | Cumulative percentage |
| Valid | Not true at all | 56 | 2,9 | 6,4 | 6,4 |
|  | Mostly not true | 281 | 14,8 | 32,3 | 38,8 |
|  | Likely to be true | 357 | 18,8 | 41,1 | 79,9 |
|  | Totally true | 175 | 9,2 | 20,1 | 100,0 |
|  | Total | 869 | 45,8 | 100,0 |  |
| Missing data | | 1030 | 54,2 |  |  |
| Total | | 1899 | 100,0 |  |  |

| judge which behaviours are associated with higher risk of coronavirus infection? | | | | | |
| --- | --- | --- | --- | --- | --- |
|  | | Frequency | Percentage | Valid percentage | Cumulative percentage |
| Valid | Not true at all | 18 | 0,9 | 2,1 | 2,1 |
|  | Mostly not true | 136 | 7,2 | 15,7 | 17,8 |
|  | Likely to be true | 451 | 23,7 | 52,1 | 69,9 |
|  | Totally true | 261 | 13,7 | 30,1 | 100,0 |
|  | Total | 866 | 45,6 | 100,0 |  |
| Missing data | | 1033 | 54,4 |  |  |
| Total | | 1899 | 100,0 |  |  |

| judge what protective measures you can apply to prevent a coronavirus infection? | | | | | |
| --- | --- | --- | --- | --- | --- |
|  | | Frequency | Percentage | Valid percentage | Cumulative percentage |
| Valid | Not true at all | 5 | 0,3 | 0,6 | 0,6 |
|  | Mostly not true | 80 | 4,2 | 9,2 | 9,8 |
|  | Likely to be true | 473 | 24,9 | 54,3 | 64,1 |
|  | Totally true | 313 | 16,5 | 35,9 | 100,0 |
|  | Total | 871 | 45,9 | 100,0 |  |
| Missing data | | 1028 | 54,1 |  |  |
| Total | | 1899 | 100,0 |  |  |

| judge how much I am at risk for a coronavirus infection? | | | | | |
| --- | --- | --- | --- | --- | --- |
|  | | Frequency | Percentage | Valid percentage | Cumulative percentage |
| Valid | Not true at all | 19 | 1,0 | 2,2 | 2,2 |
|  | Mostly not true | 188 | 9,9 | 21,7 | 23,8 |
|  | Likely to be true | 430 | 22,6 | 49,5 | 73,4 |
|  | Totally true | 231 | 12,2 | 26,6 | 100,0 |
|  | Total | 868 | 45,7 | 100,0 |  |
| Missing data | | 1031 | 54,3 |  |  |
| Total | | 1899 | 100,0 |  |  |
| judge if I have been infected with coronavirus? | | | | | |
|  | | Frequency | Percentage | Valid percentage | Cumulative percentage |
| Valid | Not true at all | 33 | 1,7 | 3,8 | 3,8 |
|  | Mostly not true | 273 | 14,4 | 31,5 | 35,3 |
|  | Likely to be true | 383 | 20,2 | 44,2 | 79,6 |
|  | Totally true | 177 | 9,3 | 20,4 | 100,0 |
|  | Total | 866 | 45,6 | 100,0 |  |
| Missing data | | 1033 | 54,4 |  |  |
| Total | | 1899 | 100,0 |  |  |

| decide how you can protect yourself from coronavirus infection based on information in the media? | | | | | |
| --- | --- | --- | --- | --- | --- |
|  | | Frequency | Percentage | Valid percentage | Cumulative percentage |
| Valid | Not true at all | 8 | 0,4 | 0,9 | 0,9 |
|  | Mostly not true | 97 | 5,1 | 11,1 | 12,1 |
|  | Likely to be true | 487 | 25,6 | 55,9 | 68,0 |
|  | Totally true | 279 | 14,7 | 32,0 | 100,0 |
|  | Total | 871 | 45,9 | 100,0 |  |
| Missing data | | 1028 | 54,1 |  |  |
| Total | | 1899 | 100,0 |  |  |

| follow instructions from your doctor or pharmacist regarding how to handle the coronavirus situation? | | | | | |
| --- | --- | --- | --- | --- | --- |
|  | | Frequency | Percentage | Valid percentage | Cumulative percentage |
| Valid | Not true at all | 13 | 0,7 | 1,5 | 1,5 |
|  | Mostly not true | 78 | 4,1 | 9,0 | 10,5 |
|  | Likely to be true | 480 | 25,3 | 55,4 | 65,9 |
|  | Totally true | 296 | 15,6 | 34,1 | 100,0 |
|  | Total | 867 | 45,7 | 100,0 |  |
| Missing data | | 1032 | 54,3 |  |  |
| Total | | 1899 | 100,0 |  |  |

| use information the doctor gives you to decide how to handle an infection with coronavirus? | | | | | |
| --- | --- | --- | --- | --- | --- |
|  | | Frequency | Percentage | Valid percentage | Cumulative percentage |
| Valid | Not true at all | 13 | 0,7 | 1,5 | 1,5 |
|  | Mostly not true | 54 | 2,8 | 6,2 | 7,7 |
|  | Likely to be true | 497 | 26,2 | 57,1 | 64,8 |
|  | Totally true | 307 | 16,2 | 35,2 | 100,0 |
|  | Total | 871 | 45,9 | 100,0 |  |
| Missing data | | 1028 | 54,1 |  |  |
| Total | | 1899 | 100,0 |  |  |

| use media information to decide how to handle an infection with coronavirus? | | | | | |
| --- | --- | --- | --- | --- | --- |
|  | | Frequency | Percentage | Valid percentage | Cumulative percentage |
| Valid | Not true at all | 15 | 0,8 | 1,7 | 1,7 |
|  | Mostly not true | 75 | 3,9 | 8,6 | 10,3 |
|  | Likely to be true | 504 | 26,5 | 57,9 | 68,2 |
|  | Totally true | 277 | 14,6 | 31,8 | 100,0 |
|  | Total | 871 | 45,9 | 100,0 |  |
| Missing data | | 1028 | 54,1 |  |  |
| Total | | 1899 | 100,0 |  |  |

| to behave in a way to avoid infecting others? | | | | | |
| --- | --- | --- | --- | --- | --- |
|  | | Frequency | Percentage | Valid percentage | Cumulative percentage |
| Valid | Not true at all | 12 | 0,6 | 1,4 | 1,4 |
|  | Mostly not true | 92 | 4,8 | 10,6 | 12,0 |
|  | Likely to be true | 458 | 24,1 | 52,6 | 64,6 |
|  | Totally true | 308 | 16,2 | 35,4 | 100,0 |
|  | Total | 870 | 45,8 | 100,0 |  |
| Missing data | | 1029 | 54,2 |  |  |
| Total | | 1899 | 100,0 |  |  |
