# Supplementary material for: Associations between Polish school principals’ health literacy and implementation of the Health Promoting School approach during the COVID-19 pandemic
Source: PLoS One. 2024 Apr 2;19(4):e0301055. doi: 10.1371/journal.pone.0301055 (PMC10986982; doi:10.1371/journal.pone.0301055)
Supplement: S2 Appendix — (ZIP) [file pone.0301055.s002.zip › School principals survey - sample size.docx]

| Gender | | | | | |
| --- | --- | --- | --- | --- | --- |
|  | | Frequency | Percentage | Valid percentage | Cumulative percentage |
| Valid | Men | 198 | 10,4 | 18,0 | 18,0 |
|  | Female | 901 | 47,4 | 82,0 | 100,0 |
|  | Total | 1099 | 57,9 | 100,0 |  |
| Missing data | | 800 | 42,1 |  |  |
| Total | | 1899 | 100,0 |  |  |

| Age range | | | | | |
| --- | --- | --- | --- | --- | --- |
|  | | Frequency | Percentage | Valid percentage | Cumulative percentage |
| Valid | 30-44 | 158 | 8,3 | 14,5 | 14,5 |
|  | 45-54 | 499 | 26,3 | 45,9 | 60,4 |
|  | >55 | 430 | 22,6 | 39,6 | 100,0 |
|  | Total | 1087 | 57,2 | 100,0 |  |
| Missing data | | 812 | 42,8 |  |  |
| Total | | 1899 | 100,0 |  |  |

| Type of school | | | | | |
| --- | --- | --- | --- | --- | --- |
|  | | Frequency | Percentage | Valid percentage | Cumulative percentage |
| Valid | primary school | 810 | 42,7 | 78,6 | 78,6 |
|  | secondary school | 220 | 11,6 | 21,4 | 100,0 |
|  | Total | 1030 | 54,2 | 100,0 |  |
| Missing data | | 869 | 45,8 |  |  |
| Total | | 1899 | 100,0 |  |  |

| Respondent position | | | | | |
| --- | --- | --- | --- | --- | --- |
|  | | Frequency | Percentage | Valid percentage | Cumulative percentage |
| Valid | School principal | 810 | 42,7 | 74,2 | 74,2 |
|  | Vice-principal | 281 | 14,8 | 25,8 | 100,0 |
|  | Total | 1091 | 57,5 | 100,0 |  |
| Missing data | | 808 | 42,5 |  |  |
| Total | | 1899 | 100,0 |  |  |
